# Estimating Youth Locomotion Ground Reaction Forces Using an Accelerometer-Based Activity Monitor

**DOI:** 10.1371/journal.pone.0048182

**Published:** 2012-10-25

**Authors:** Jennifer M. Neugebauer, David A. Hawkins, Laurel Beckett

**Affiliations:** 1 Biomedical Engineering Graduate Group, University of California Davis, Davis, California, United States of America; 2 Department of Neurobiology, Physiology & Behavior, University of California Davis, Davis, California, United States of America; 3 Department of Public Health Sciences, University of California Davis, Davis, California, United States of America; University of Zaragoza, Spain

## Abstract

To address a variety of questions pertaining to the interactions between physical activity, musculoskeletal loading and musculoskeletal health/injury/adaptation, simple methods are needed to quantify, outside a laboratory setting, the forces acting on the human body during daily activities. The purpose of this study was to develop a statistically based model to estimate peak vertical ground reaction force (pVGRF) during youth gait. 20 girls (10.9±0.9 years) and 15 boys (12.5±0.6 years) wore a Biotrainer AM over their right hip. Six walking and six running trials were completed after a standard warm-up. Average AM intensity (g) and pVGRF (N) during stance were determined. Repeated measures mixed effects regression models to estimate pVGRF from Biotrainer activity monitor acceleration in youth (girls 10–12, boys 12–14 years) while walking and running were developed. Log transformed pVGRF had a statistically significant relationship with activity monitor acceleration, centered mass, sex (girl), type of locomotion (run), and locomotion type-acceleration interaction controlling for subject as a random effect. A generalized regression model without subject specific random effects was also developed. The average absolute differences between the actual and predicted pVGRF were 5.2% (1.6% standard deviation) and 9% (4.2% standard deviation) using the mixed and generalized models, respectively. The results of this study support the use of estimating pVGRF from hip acceleration using a mixed model regression equation.

## Introduction

For many studies, quantifying the forces acting on and within the body during daily living is of great interest. This allows further understanding of the relationships between loading of musculoskeletal structures and structure development, injury, and adaptation (e.g. bone development and physical activity correlates [1], post surgical weight bearing asymmetry [2]). To facilitate such studies, simple methods are needed that can be employed outside a laboratory setting to quantify various forces acting on and within the human body during daily activities. Accelerometer-based activity monitors (referred to as AMs throughout), pedometer-sized devices worn on the hip, wrist or ankle, have been used to quantify metabolic expenditure [3]–[8], but a less explored and potentially useful application for AMs is to estimate forces acting on the body. A few investigators explored the use of AMs to estimate ground reaction forces and obtained results warranting further investigation [9]–[11].

Knowledge of the ground reaction forces (GRFs) acting on the foot during locomotion is essential for investigations into the interaction between the loading of lower extremity structures during gait and the development, injury and adaptation of these structures. GRFs can be quantified in the laboratory using force plates, but quantification during daily activities outside of the laboratory is not practical using force plates. Methods to calculate GRFs using pressure insoles have been investigated to quantify GRFs outside of a laboratory but result in attenuated peak forces compared to those measured with a force plate [12]–[13]. Additionally, data collection over multiple days outside of the laboratory is not currently possible with pressure insoles. AMs may provide a means to estimate peak vertical GRFs (pVGRFs) over multiple days of daily activities. pVGRF is the component of ground reaction forces with the largest magnitude during the stance phase of gait. While previous reports of the link between pVGRF and injury have been mixed [14], assessment of the pVGRF along with the frequency of its occurrence would provide a ‘snapshot’ of a subject's pVGRF loading profile over a period of time, possibly providing more insight into the interaction between musculoskeletal structural/material changes and loading, as well as overuse injury development.

Studies by Janz, et al. [11], Garcia, et al. [10], and Rowlands and Stiles [15] established the correlation between average peak ground reaction forces (GRF) during the stance phase of gait and AM counts, but did not determine if AM data could predict GRF during various walking and running speeds. Janz, et al. [11] concluded the Computer Science and Applications (CSA) AM was useful for measuring ambulation in youth; however, they also state that the CSA was designed for accelerations between 0.05 to 2 g. Accelerations sustained while running have been reported as high as 5–6 g [16]. Garcia, et al. [10] demonstrated through simple regression a linear relationship between average vertical GRF and Biotrainer AM activity counts. In this study however, Biotrainer AM activity counts were determined from 10 minute epochs, and only one walking and one jogging speed were used. Exercise bouts of interest may last less than 10 minutes, such as high intensity, short duration efforts, and therefore it is unclear how accurate the approach by Garcia, et al. [10] would be for estimating average vertical GRF during variable speed activities. Additionally, a leveling off of AM counts with increasing running speeds that does not occur with walking has previously been shown [9]. The differing relationship when walking compared with running between AM counts and speed challenges the validity of using only one walking and one running speed. Testing at multiple walking and running speeds as well as accounting for the differing responses of pVGRF with increasing AM acceleration for walking and running would better characterize the relationship between pVGRF and AM acceleration while walking and running.

The purpose of this study was to develop a statistically based model to estimate pVGRF from Biotrainer AM acceleration in girls 10–12 and boys 12–14 years of age (These age ranges represent the average age ranges during which peak height velocity occurs for girls and boys [17]–[20]) for walking and running and to evaluate the accuracy of the pVGRF estimates. The Biotrainer AM, a pedometer-sized biaxial accelerometer, was selected for investigation due to five distinct characteristics of this AM compared with other commercially available AMs at the start of this study (first monitors purchased in 2009): (1) relatively low cost, (2) short epoch duration (15 seconds), (3) storage capacity (five days using the shortest epoch duration), (4) capacity to measure accelerations as high as 7 g, and (5) fastest sampling rate (40 Hz) in a monitor that also provided output in acceleration. The goals of the study were achieved by developing a repeated measures mixed effects regression model that included subject specific random effects. A more general repeated measures regression model that did not include subject specific random effects was also developed.

## Methods

### Ethics

The study was approved by the University of California, Davis Institutional Review Board and prior to testing, written informed parental consent and subject assent (12 years of age or older) were obtained.

### Participants

20 girls between 10 and 12 years of age and 15 boys between 12 and 14 years of age were recruited. These age ranges represent the average age ranges during which peak height velocity occurs for girls and boys [17]–[20] and are of interest for various studies being conducted in our laboratory [21]. All testing was conducted in the Human Performance Laboratory at UC Davis.

### Description of Procedures

After informed consent and assent were obtained, the subject's height and mass were determined to the nearest 0.5 cm and 0.1 kg, respectively. Body mass index (BMI) was calculated for all subjects (mass (kg) divided by height squared (m)). Centered mass (cmass) for each subject was calculated. Cmass was defined as the difference between the subject's mass and a reference average mass. The reference masses used were determined from the 50th percentile mass for boys (45 kg) and girls (37 kg) of the study population mean age for boys and girls based on Centers for Disease Control clinical growth charts [22]. A boy with a cmass of 0.0 kg has a mass of 45.0 kg, for example. Similarly, a girl with a cmass of −7.0 kg had a mass of 30 kg. Cmass created an independent variable to be used in the regression models that allowed both prediction for a standard reference child, typical in mass by sex for this age group, and estimation of the modifying effect for children of lighter or heavier mass than average.

Subjects wore a randomly assigned (assignments made prior to the start of the study) Biotrainer AM (5×7.5 cm weighing 0.055 kg with the battery, using Biotrainer Pro 6.130 software; IM Systems, Baltimore, MD, USA) secured on their waistband over the most lateral aspect of the iliac crest of their right hip. Ten different monitors were used for this study. The monitor was initialized to record 15 second epochs using a 40 Hz sampling rate and a gain of 40. The Biotrainer AM outputs the average peak resultant (vertical and anteroposterior axes) acceleration (g; 1 g = 9.807 m/s^2^) during the specified epoch length (15 seconds for this study).

Subjects were oriented to the protocol and allowed to practice until both the subject and investigator were confident in the subject's ability to successfully complete trials. Subjects completed walking trials and running trials along a 90 m path that included a force plate (Kistler Corporation, Model 9281B (40×60 cm), Amherst, NY, USA) about 6 m from the starting point and four turns along two hallways. Force plate data were collected using a custom Labview data acquisition program, sampled at 1000 Hz. The AM and data collection computer were synchronized with an atomic clock that was used to identify the start time of each trial.

Walking and running trials were performed in a randomly assigned order following a standard warm-up that consisted of three full-length practice trials. Trials were initiated on a 15 second increment in order to identify the epochs for each trial, and lasted at least 45 seconds (minimum of three epochs per trial). Subjects were informed of the force plate location, but encouraged to look at a sight target located at eye level in front of them. Locomotion speed was determined using electronic timing gates located two meters around the force plate and synchronized with force plate data acquisition. Speed was measured as the subject crossed the force plate, but was not measured throughout the trial. To help ensure a consistent speed during the trial, a trained research assistant walked/ran along side the subject throughout the trial. Trials in which the subject appeared to alter their gait in any way to successfully contact the plate (defined as full right foot contact with the plate) were not included in the analysis.

For each trial, the average acceleration (g) of 2 epochs (30 seconds total) and pVGRF (N) during the support phase on the force plate were determined. Trials with an AM acceleration difference greater than 10% between the two epochs were assumed to be indicative of varied speed or gait during the trial and were therefore not included in the analysis. A total of 12 trials from each subject (6 walk, and 6 run) were used for statistical analysis.

### Statistical methods

Means and standard deviations were determined for subject demographics and differences between sexes assessed using t-tests with significance defined at p<0.05. Differences in pVGRF, speed, and AM acceleration between sex and type of locomotion (walk and run) were determined using repeated measures ANOVA with subject as a random effect and fixed effects of sex (between-subject factor) and type of locomotion (walk or run, within-subjects factor). A prediction model was developed for pVGRF using repeated measures mixed effects regression [23]. Core hypothesized predictors were sex and cmass, (between-child predictor), and type of locomotion (walking or running), AM acceleration, and the interaction of AM acceleration and type of locomotion (within-child factors). We also checked to determine whether the relationship might be confounded by height or by the specific AM worn. Random effects allowed for between-subject differences in overall level and in rate of increase of pVGRF with AM acceleration and within-subject variation from an overall error term. Significance was defined at p<0.05 (R, R Foundation for Statistical Computing, Austria). Once non-significant confounding effects were eliminated from the model, significance of the random effects was determined by hierarchical fitting with and without random effects. Significant results (p<0.05) indicated the subject-specific slope and intercept were both needed. The model was powered at β = 0.80 [24].

Additionally, a more generalized model was developed that did not include subject specific random effects. The generalized model was developed to be applicable to studies where a subject's random effects could not be determined. For larger, population based studies, the ability to conduct a laboratory calibration session for every subject may not be feasible, precluding the use of subject-specific effects in a regression model. The fixed effects for the generalized model included between- and within-subjects factors that could be easily known or measured on subjects in a larger population study: sex, mass, height (between-subjects) and AM acceleration, type of locomotion, and an interaction between AM acceleration and type of locomotion (within subjects). A final model eliminated non-significant predictors.

Based on the final mixed effects and generalized regression models, predicted pVGRFs were compared with the actual pVGRFs for each subject. Results were summarized to assess both the factors predicting the overall population mean pVGRFs and the accuracy of those predictions for individual subjects, reflected in individual variation from the means. Model assumptions (linearity of relationships, normality and homoscedasticity of residuals) were checked via residual analysis (Bland-Altman plots, Q-Q plots, and summary diagnostics) to ensure that both the prediction equation and the single-number summaries of accuracy of prediction gave accurate representations of the full dataset.

## Results

Age, height, and mass all significantly differed between boys and girls (Table 1). BMI and cmass did not differ significantly between boys and girls. In general, AM acceleration increased with increasing speed and pVGRF increased with increasing AM acceleration. pVGRF (N) differed (p<0.05) between boys and girls during both walking and running trials. Speed and AM acceleration did not differ between sexes for walking (p = 0.455 and p = 0.433, respectively) or for running (p = 0.206 and p = 0.738, respectively) (Table 2).

**Table 1:** Subject demographics for study population.

	Boys	Girls
n	15	20
Age (years)	12.5±0.6	10.9±0.8 *
Height (m)	1.65±0.11	1.55±0.09 *
Mass (kg)	54.9±11.7	45.6±11.5 *
BMI (kg/m^2^)	20.14±2.97	19.13±3.40
Centered mass (kg)	8.6±11.7	9.9±11.5

Mean ± one standard deviation are reported.

* Significant (p<0.05) difference between boys and girls.

**Table 2:** Summary of walking and running trials.

	Walking Trials: Average (Range)		Running Trials: Average (Range)	
	Boys	Girls	Boys	Girls
Speed (m/s)	1.55 (0.90–2.26)	1.59 (0.82–2.29)	2.83 (2.57–3.88)	2.98 (2.17–3.83)
AM Acceleration (g)	1.69 (0.45–3.45)	1.60 (0.50–3.70)	4.58 (2.75–6.05)	4.48 (2.00–6.30)
pVGRF (N)	713.6 (419.0–1185.3)	608.2 * (336.7–1288.9)	1301.1 (804.3–1929.5)	1110.2 * (712.3–1987.2)

Average speed, AM acceleration, and pVGRF for boys and girls in both walking and running trials. Average for all subjects are reported and the range of values in parentheses. Standard deviations are not reported due to the repeated measures for each subject.

* Significant (p<0.05) difference between boys and girls.

The distribution of residuals was initially noted to be non-Gaussian, so natural log transformation of pVGRF was used as the outcome in all regression models. Model diagnostics from the transformed data showed no deviations from assumptions and no pattern of systematic error in the residuals (Bland-Altman plot (for the mixed effects model) showed upper and lower 95% agreement limits of 125.43 and −130.98 N, respectively, with a bias of −4.96 N (115.80 N standard deviation)).

In the mixed effects model (Equation 1), ln(pVGRF) was significantly lower for girls compared to boys and higher for running compared to walking (Table 3). Greater AM acceleration was associated with significantly greater ln(pVGRF), although the effects were significantly lower for running than for walking, so that the effect was primarily for walking speeds. In addition, greater mass was associated with significant increases in pVGRF, but there was no significant association with height or specific AM worn by the subject (p = 0.661 and 0.860, respectively). Between-subject random variation in overall level and in the effects of AM acceleration was also statistically significant (p<0.001). The 95% confidence intervals for the variation attributable to random effects intercept (A_i0_) were 0.086 to 0.155, or about 8%–16% variation from child to child on the original pVGRF scale, and for the slope (A_i1_) were 0.022 to 0.043, or about 2–4%. The final model is given by Equation 1.

**Table 3:** Coefficients for the mixed and generalized model.

	Mixed Effects Model (Equation 1)		Generalized Model (Equation 2)	
	β	SE	α	SE
Intercept	6.031§	0.035	5.387§	0.032
AM acceleration (g)	0.210§	0.011	0.159§	0.013
Centered mass (kg)	0.016§	0.001	–	–
Mass (kg)	–	–	0.016§	0.001
Type of locomotion (walk/run where walk = 0 and run = 1)	0.647§	0.049	0.799§	0.046
AM acceleration*run interaction	−0.141§	0.014	−0.142§	0.016
Sex (boy/girl, where boy = 0 and girl = 1)	−0.138§	0.033	–	–

β and α coefficients (Equation 1 and Equation 2, respectively) along with their standard errors (SE) are reported.

§ p<0.001.



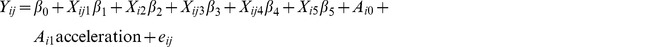
(1)where: Y_ij_  =  log-transformed pVGRF (ln(N)) for subject i, trial j. X_ij1_  =  AM acceleration (g). X_i2_  =  centered mass (kg). X_ij3_  =  type of locomotion (walk/run, where walk  = 0 and run  = 1). X_ij4_  = AM acceleration*type of locomotion interaction (g). X_i5_  =  sex (boy/girl, where boy  = 0 and girl  = 1). β  =  coefficient associated with respective fixed effect. A_i0_  =  overall tendency for child i to be different from other subjects. A_i1_acceleration  =  differential response to increasing acceleration for subject i. e_ij_  =  error in trial j for subject i.

A one g increase in AM acceleration was associated with a 19% increase in pVGRF at a walking gait, holding all other factors constant. Running gait overall was associated with over a two-fold increase in pVGRF compared to walking, but increasing the running AM acceleration by one g was associated with only a 7% increase in pVGRF, which was significantly less than the effect of increasing the walking AM acceleration. Each added kg of mass was associated with a 1.3% increase in pVGRF. The effect of increased mass was not found to differ at higher walking or running speeds, or between boys and girls.

**Figure 1:**
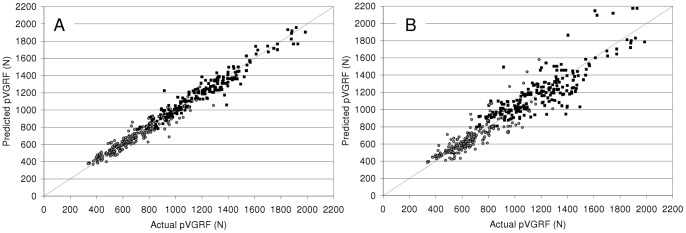
Comparison of actual pVGRF and predicted pVGRF. Panel A uses the mixed effects model (Equation 1) and Panel B uses the generalized model (Equation 2). The linearity of the relationship using both the mixed effects (r^2^ = 0.967, p<0.001) and the generalized model (r^2^ = 0.877, p<0.001) illustrates the predictive ability of the models.

Based on the final model, predicted pVGRFs were determined and compared with actual pVGRFs (Figure 1). For all subjects, using the mixed effects model, the average absolute difference between actual and predicted pVGRF (N) was 5.2% of the actual pVGRF with 1.6% standard deviation. This suggests that the pVGRF measurements across a range of speeds are well described by the model, both for boys and girls and for running and walking, provided that child-specific overall differences are incorporated into the model.

**Figure 2:**
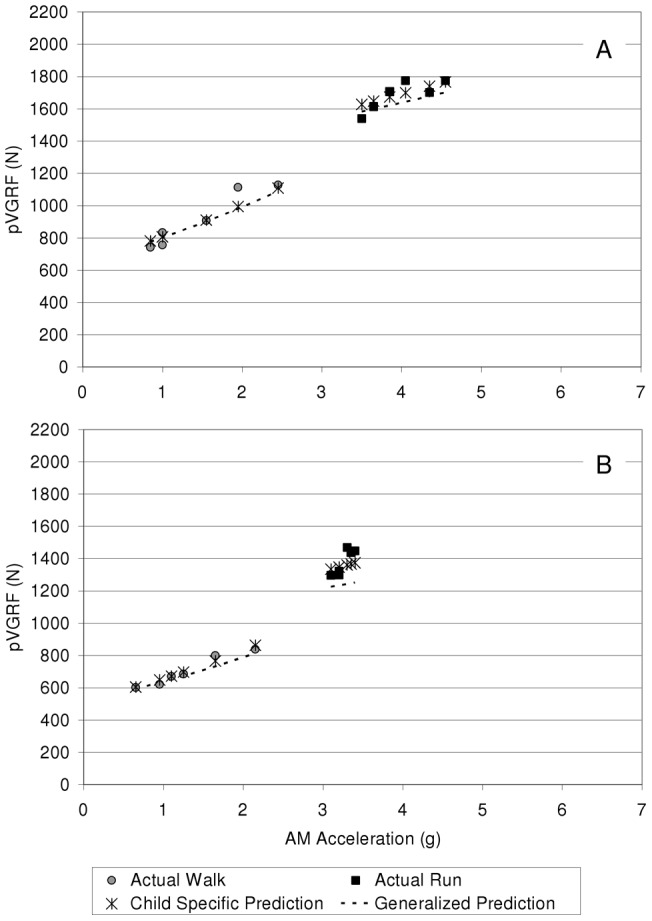
Predicted and actual pVGRF for two representative subjects. Child specific prediction (includes child specific random effects; using mixed effects model (Equation 1)), generalized prediction (no random effects; Equation 2), and actual pVGRF for walking and running are shown. Panel A illustrates a subject with increasing pVGRF as AM acceleration increases (running speeds ranged from 2.56–3.35 m/s for this subject) while Panel B illustrates a subject with a more clustered pVGRF around a similar AM acceleration for the range of running speeds used (running speeds ranged from 2.55–3.66 m/s for this subject).

For all subjects, as walking speed increased, pVGRF and AM acceleration both increased. For most subjects (28 of 35), as running speed increased, both AM acceleration and pVGRF increased as well (representative subject shown in Figure 2, A). For the remaining 7 subjects (mass 51.0±5.9 kg, height 1.58±0.02 m, cmass −1.0±5.9 kg), as running speed increased, pVGRF was clustered around a small range of AM acceleration (representative subject shown in Figure 2, B).

The generalized model (e.g. the model that did not include random effects) assessed variation from the overall prediction and treated child-specific variation as simply part of the unexplained error. This model demonstrated similar relationships as found in the mixed effects model between activity-specific predictors, mass, and log transformed pVGRF (Equation 2 and Table 3). Sex (p = 0.501) and height (p = 0.291) were not significant factors.
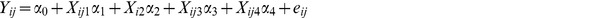
(2)where: Y_ij_  =  log-transformed pVGRF ln((N)) for subject i, trial j. X_ij1_  =  AM acceleration (g). X_i2_  =  mass (kg). X_ij3_  =  type of locomotion (walk/run, where walk  = 0 and run  = 1). X_ij4_  =  AM acceleration*type of locomotion interaction (g). α  =  coefficient associated with respective fixed effect. e_ij_  =  error in trial j for subject i.

Predictions based on the generalized model (Equation 2) with no subject specific random effects resulted in larger percent differences between the actual and predicted pVGRF compared to the mixed effects model (Figure 2). The average absolute difference between the actual and predicted pVGRF using the generalized model was 9% for all subjects (4.2% standard deviation). The doubling of the error in prediction reflects the fact that the generalized model does not incorporate the important, significant differences that exist between children and that are not explained by measured characteristics such as sex, height, or mass.

## Discussion

A statistically based model was developed to predict pVGRF from Biotrainer AM acceleration for girls 10 to 12 and boys 12 to 14 years of age for walking and running. While numerous studies have demonstrated the use of AMs to estimate metabolic expenditure [25]–[26], few to date investigated the use of AMs to predict ground reaction forces [9]–[11]. AMs provide a potential portable means to assess skeletal impact loads during daily living activities over the course of multiple days [15], something that cannot be done in lab-based studies. However, to use AMs for this purpose, appropriate algorithms must be developed to convert AM measures into measures of ground reaction forces and/or musculoskeletal loading. The goal of this study was to develop such an algorithm to scale the output data of a Biotrainer AM to pVGRF for walking and running youth.

Logarithmically transformed pVGRF was well predicted using a mixed effects model to account for the repeated measures from each subject. In this study, pVGRF had a positive, increasing relationship with AM acceleration, similar to previously reported results [10]–[11]. Body mass was a significant predictor of pVGRF, consistent with body weight as a predictor as reported by Janz, et al. [11]. The average mass for boys and girls in the study was larger than the CDC reported averages as evidenced by the positive centered masses. The 50th percentile mass for boys and girls of the mean age of the study population is a population average and therefore some deviation from this can be expected. Girls in this study were approximately 82^nd^ percentile for mass and greater than the 95^th^ percentile for height and boys were approximately 85^th^ percentile for mass and 95^th^ percentile for height. Sex, though not considered in previous studies, was a significant predictor when subject specific random effects were included (Equation 1), with girls having a lower slope than boys. The specific monitor worn did not lead to differing results.

The theoretical basis that motivated this investigation, to develop a model to estimate peak ground reaction force from hip acceleration, is the fundamental equations of motion that describe gait. Solving the equations of motion for ground reaction force reveals that GRF is a function of subject mass, moments about the ankle, knee, and hip, inertial moments about the ankle, knee, and hip, segmental masses and accelerations of the foot, shank, and thigh. For a given subject, several variables in this relationship would remain constant such as subject mass, segment moments of inertia, and segment center of mass locations. Other quantities are directly related to locomotion speed, such as segment linear and angular accelerations. Thus, it is reasonable to assume that GRF for specific types of movement could be predicted from hip acceleration data. Additional variables could be included in the regression model to improve the prediction of pVGRF, such as locomotion speed, subject specific estimates of limb mass, angular velocities, or moments about joint centers. For the purposes of this model, we did not include locomotion speed because it would not be easily determined outside of a laboratory. Previous studies have reported the relationship between speed and ground reaction force [27]–[28]. Given the small error in predicted versus actual pVGRF using the models develop here, addition of these variables would likely not greatly improve the predictive capabilities of the model during steady state efforts.

Previous studies investigated the use of AMs to predict ground reaction force, but with a minimal range of walking and running speeds. Studies by Garcia, et al. and Janz, et al. [10]–[11] based their regressions on one walking and one running speed, and two walking and one running speeds, respectively. Limited locomotion speeds allow for minimal determination of the relationship of the AM acceleration with ground reaction force and assume linearity with a constant slope across the entire range including both walking and running. Differences for walking and running in the relationship between AM counts and ambulation speed (activity counts are a device specific arbitrary unit that relate the frequency of accelerations incurred during the specific epoch duration) [9] has been previously reported. The study presented here utilized six different walking speeds and six different running speeds to allow separate estimation of the relationship between AM acceleration and vertical ground reaction force for running and walking gaits. Analyses demonstrated a difference between slopes for running and walking. Increased AM acceleration was associated with higher pVGRF as walking gait varied. For running gait, as AM acceleration increased, pVGRF had a lower slope than in walking gait. The differing relationship between AM acceleration and pVGRF during walking and running gaits confirm the inadequacy of a combined linear regression model that does not include sufficient data to account for differences in slopes for walking and running.

This study not only provided a good predictive model for pVGRF, but also characterized variations between subjects. Subjects differed not only in the magnitude of their overall pVGRF, but also in the amount pVGRF changed as gait speed increased. For walking, all subjects showed an increase in pVGRF with higher AM acceleration, with most differences between predicted and actual pVGRF within 11% of the fitted mean. The individual differences in slope were more striking in the running trials. For some subjects (7 of 35), as running speed increased, AM intensity did not increase for the range of speeds tested, resulting in a clustered pVGRF pattern. These results are similar to previously reported leveling off of AM counts (study conducted using CSA AM) at higher running speeds (running speeds used ranged from 2.2 m/s to 5.5 m/s) [9]. While the speeds used by Brage, et al. were faster than currently used, the AM counts started leveling off at approximately 2.8 m/s which is within the range of speeds currently used (2.3–3.8 m/s). For the seven subjects for whom the clustering pVGRF was observed, no remarkable differences in their speeds were observed relative to the other 28 subjects. This lack of variation in AM acceleration as running speed increased could be due to the age of the subjects and their still developing motor patterns or a normal variation in gait mechanics. Brage, et al. [9] concluded this observation was due to the relatively constant vertical accelerations that occur during running. Further investigation of the kinematics and kinetics may help explain the similar AM accelerations for the running speeds tested in some subjects.

While the study provided insight into the magnitude of between-child variation (random effects confidence intervals), neither the source nor the clinical significance of these differences is currently known. Within-child variation was small, suggesting that some consistent features either of the child's physical make-up or performance are likely to account for differences in musculoskeletal loading during exercise. Furthermore, the differences in musculoskeletal loading may have clinical significance, such as increased injury risk, that has yet to be explored. The results of this study in combination with future specific injury risk investigations could further clarify the significance of the between-child variations observed.

This model provides practical information for two different areas of application. For larger, population based studies, the ability to conduct a laboratory calibration session for every subject may not be feasible, precluding the use of subject-specific calibrated estimates. The generalized model provides an overall prediction of mean pVGRF trajectories for walking and running gaits, calibrated for subject mass. An extension of the model, making use of the random effects estimates, provides an estimate of the likely range of pVGRF for individual subjects. While the average absolute difference in predicted pVGRF compared with actual pVGRF was 9% for the generalized model, five subjects had larger errors in predicted pVGRF (17.5% average absolute difference). Of possible interest is that four of the five subjects with the larger errors in predicted pVGRF had clustered running pVGRFs. As previously mentioned, the clinical significance of these individual differences is not known. A 17.5% error in prediction may represent a clinically significant difference in the skeletal loading for a particular subject whereas for another subject the larger percent differences may have no implications. Further investigation into the sources and the implications of the individual variations would clarify the acceptable differences in predicting pVGRF. A strength of the mixed models is the quantification of between- and within-individual variation, which provides key information for design of future studies to try to understand the reasons for this variation.

### Limitations

While this study provides insight to the use of the AM to estimate pVGRF, several limitations must be appreciated. First, as previously demonstrated by other authors, jumping activities are not accurately represented by the AM. If loads sustained during an activity that combines walking, running, and jumping, such as a basketball game, are of interest, further investigation of the effects of these variable activities on AM acceleration should be investigated. Secondly, although the shortest available time epochs were used in this study (15 seconds) youth can change their activity acceleration in seconds. The results from this study are specific to steady state efforts. If a subject were to run fast for 5 seconds, run slow for 5 seconds, and rest for 5 seconds, the average AM acceleration for that 15 seconds of activity would not be representative of the three activity levels that actually occurred within the epoch. While the average acceleration of the 15 second epoch may be of interest, if the specific loading incurred during the 5 seconds of sprinting is of interest, researchers should choose a monitor that outputs raw data or smaller epoch durations. With the recent introduction of monitors that have shorter than 15 second epochs as well as those that output raw acceleration data, this limitation may be eliminated in the near future. Ground reaction force was determined once during each 30 second trial. While more contacts with the force plate would be ideal to provide an average pVGRF, the AM provided an estimate of the consistency of effort during the trial. AM epochs that differed by greater than 10% were not included in the analysis. Future work that combines varying walking and running speeds using a force plate instrumented treadmill would provide for further confirmation of the pVGRFs throughout the duration of the trial. Thirdly, only pVGRF was investigated in this study to demonstrate proof-of-concept, but current methods could be extended to include the resultant GRFs or other force components. Lastly, testing of additional subjects across a wider age range would allow for a more general youth model to be developed.

### Summary

A mixed effects repeated measures regression model to predict pVGRF from Biotrainer AM acceleration was developed for girls 10 to 12 and boys 12 to 14 years of age for walking and running. pVGRF can be estimated using a model that includes the fixed effects of AM acceleration, centered mass, type of gait, and sex, and random effects of subject specific responses to increasing AM acceleration. For some subjects, pVGRF minimally increased as running speed increased. A generalized model was also developed that is applicable to larger, population based studies where lab testing to determine subject specific effects is not feasible.

## References

[1] Janz KF, Gilmore JM, Levy SM, Letuchy EM, Burns TL, et al (2007) Physical activity and femoral neck bone strength during childhood: the Iowa Bone Development Study. Bone 41: 216–22. 17560839 10.1016/j.bone.2007.05.001 PMC2002473

[2] Christiansen CL, Bade MJ, Judd DL (2011) Weight-bearing asymmetry during sit-stand transitions related to impairment and functional mobility after total knee arthroplasty. Arch Phys Med Rehabil 92: 1624–1629. 21839986 10.1016/j.apmr.2011.05.010 PMC4526185

[3] Freedson P, Pober D, Janz KF (2005) Calibration of accelerometer output for children. Med Sci Sports Exerc 37: S523–530. 16294115 10.1249/01.mss.0000185658.28284.ba

[4] Freedson PS, Melanson E, Sirard J (1998) Calibration of the Computer Science and Applications, Inc. accelerometer. Med Sci Sports Exerc 30: 777–781. 9588623 10.1097/00005768-199805000-00021

[5] Rowlands AV, Eston RG (2005) Comparison of accelerometer and pedometer measures of physical activity in boys and girls, ages 8–10 years. Res Q Exerc Sport 76: 251–257. 16270702 10.1080/02701367.2005.10599296

[6] Trost SG, Pate RR, Sallis JF, Freedson PS, Taylor WC, et al (2002) Age and gender differences in objectively measured physical activity in youth. Med Sci Sports Exerc 34: 350–355. 11828247 10.1097/00005768-200202000-00025

[7] Welk GJ, Almeida J, Morss G (2003) Laboratory calibration and validation of the Biotrainer and Actitrac activity monitors. Med Sci Sports Exerc 35: 1057–1064. 12783056 10.1249/01.MSS.0000069525.56078.22

[8] Johannsen DL, Calabro MA, Stewart J, Franke W, Rood JC, et al (2010) Accuracy of armband monitors for measuring daily energy expenditure in healthy adults. Med Sci Sports Exerc 42: 2134–40. 20386334 10.1249/MSS.0b013e3181e0b3ff

[9] Brage S, Wedderkopp N, Franks PW, Andersen LB, Froberg K (2003) Reexamination of validity and reliability of the CSA monitor in walking and running. Med Sci Sports Exerc 35: 1447–1454. 12900703 10.1249/01.MSS.0000079078.62035.EC

[10] Garcia AW, Langenthal CR, Angulo-Barroso RM, Gross MM (2004) A comparison of accelerometers in school-age children for predicting energy expenditure and vertical ground reaction force. Meas Phys Educ Exerc Sci 8: 119–144.

[11] Janz KF, Rao S, Baumann HJ, Schultz JL (2003) Measuring children's vertical ground reaction forces with accelerometry during walking, running, and jumping: The Iowa Bone Development Study. Pediatr Exerc Sci 15: 34–43.

[12] Barnett S, Cunningham JL, West S (2001) A comparison of vertical force and temporal parameters produced by an in-shoe pressure measuring system and a force platform. Clin Biomech 16: 353–357. 10.1016/s0268-0033(01)00026-2 11358623

[13] Low DC, Dixon SJ (2010) Footscan pressure insoles: accuracy and reliability of force and pressure measurements in running. Gait Posture 32: 664–666. 20813530 10.1016/j.gaitpost.2010.08.002

[14] Zadpoor AA, Nikooyan AA (2011) The relationship between lower-extremity stress fractures and the ground reaction force: a systematic review. Clin Biomech 26: 23–28. 10.1016/j.clinbiomech.2010.08.005 20846765

[15] Rowlands AV, Stiles VH (2012) Accelerometer counts and raw acceleration output in relation to mechanical loading. J Biomech 45: 448–454. 22218284 10.1016/j.jbiomech.2011.12.006

[16] Crowell HP, Milner CE, Hamill J, Davis IS (2010) Reducing impact loading during running with the use of real-time visual feedback. J Orthop Sports Phys Ther 40: 206–213. 20357417 10.2519/jospt.2010.3166

[17] Berkey CS, Dockery DW, Wang X, Wypij D, Ferris B (1993) Longitudinal height velocity standards for U.S. adolescents. Stat Med 12: 403–414. 8456221 10.1002/sim.4780120321

[18] Thissen D, Bock D, Wainer H, Roche AF (1976) Individual growth in stature: a comparison of four growth studies in the U.S.A. Ann Hum Biol. 3: 529–542. 10.1080/03014467600001791 999229

[19] Philippaerts RM, Vaeyens R, Janssens M, Van Renterghem B, Matthys D, et al (2006) The relationship between peak height velocity and physical performance in youth soccer players. J Sports Sci 24: 221–230. 16368632 10.1080/02640410500189371

[20] Rauch F, Bailey DA, Baxter-Jones A, Mirwald R, Faulkner R (2004) The ‘muscle-bone unit’ during the pubertal growth spurt. Bone 34: 771–775. 15121007 10.1016/j.bone.2004.01.022

[21] Neugebauer JM, Hawkins DA (2012) Identifying factors related to Achilles tendon stress, strain, and stiffness before and after 6 months of growth in youth 10–14 years of age. J of Biomech 45: 2457–2461. 22877892 10.1016/j.jbiomech.2012.06.027 PMC3654684

[22] Kuczmarski RJ, Ogden CL, Guo SS, Grummer-Strawn LM, Flegal KM, et al. (2000) 2000 CDC Growth Charts for the United States: Methods and Development website. Available: http://www.cdc.gov/growthcharts/clinical_charts.htm#Set1. Accessed 2012 September 28.

[23] Fox J (2002) Linear Mixed Models; Appendix to An R and S-PLUS Companion to Applied Regression web site. Available: http://cran.r-project.org/doc/contrib/Fox-Companion/appendix-mixed-models.pdf. Accessed 2012 September 28.

[24] Diggle PJ, Liang KY, Zegar SL (1994) Analysis of longitudinal data. New York: Oxford University Press. 253 p.

[25] Trost SG, Ward DS, Moorehead SM, Watson PD, Riner W, et al (1998) Validity of the computer science and applications (CSA) activity monitor in children. Med Sci Sports Exerc 30: 629–633. 9565947 10.1097/00005768-199804000-00023

[26] Welk GJ, Eisenmann JC, Schaben J, Trost SG, Dale D (2007) Calibration of the Biotrainer pro activity monitor in children. Pediatr Exerc Sci 19: 145–158. 17603138 10.1123/pes.19.2.145

[27] Nilsson J, Thorstensson A (1998) Ground reaction forces at different speeds of human walking and running. Acta Physiol Scand 136: 217–27. 10.1111/j.1748-1716.1989.tb08655.x 2782094

[28] Stansfield BW, Hillman SJ, Hazlewood ME, Lawson AA, Mann AM, et al (2001) Normalized speed, not age, characterizes ground reaction force patterns in 5- to 12-year-old children walking at self-selected speeds. J Pediatr Orthop 21: 395–402. 11371828

